# Characterization of the complete chloroplast genome of *Dioscorea polystachya* Turcz.

**DOI:** 10.1080/23802359.2021.1927222

**Published:** 2021-05-17

**Authors:** Na Hu, Jianwu Gong, Biming Zhang

**Affiliations:** The Fourth Hospital of Changsha, Changsha, PR China

**Keywords:** *Dioscoreaceae*, complete chloroplast genome, *Dioscorea polystachya*, phylogenetic

## Abstract

*Dioscorea polystachya* Turcz. is an important Chinese herbal medicine and the raw material of the medicine ingrediente (Chinese yam polysaccharide). It belongs to *Dioscorea*, which has 60 species and distribute in middle and southeast of China. In this study, we sequenced the sample of *D. polystachya* based on Jiaozuo, Henan and determined its complete chloroplast genome. The length of cp genome was 152,958 bp, includes two invert repeats (IR) regions of 25,492 bp, a large single-copy (LSC) region of 83,152 bp, and a short single-copy (SSC) region of 18,822 bp. There were 131 genes, which included 85 protein coding genes, 8 rRNA, and 38 tRNA, and overall GC content covered by 37.1%. Each of *trn*K-UUU, *rps*16, *trn*G-UCC, *atp*F, *rpo*C1, *trn*L-UAA, *trn*V-UAC, *pet*B, *pet*D, *rpl*16, *rpl*2, *ndh*B, *trn*I-GAU, *trn*A-UGC, and *ndh*A genes contained an intron, *clp*P and *ycf*3 contained 2 introns. The phylogenetic position showed that *D. Polystachya* had the closest relationship with *Dioscorea alata* (MG267382) *and Dioscorea aspersa* (NC039807).

*Dioscorea polystachya* Turcz. is a temperate plant that distributes in Henan, Jiangsu, Anhui, etc., and belongs to the family of *Dioscoreaceae. Dioscoreaceae* contains 9 genus and over 600 species around worldwide (Andres et al. 2017), but in China there is only 1 genus and 60 species. *D. polystachya* is not only used as the food and herbal medicine, but also an important raw material of the medicine ingredient (Chinese yam polysaccharide, a kind of ingredient in antihypertensive) (Luo et al. 2016). *D. polystachya* base on Jiaozuo, Henan has around 0.7–1 m long and 1.5–3 cm diameter stick root, on the root there are some dark brown or rusty flecks. It was used as raw material of medicine ingredient much more than food. We can hardly find the complete chloroplast of *D. polystachya* based here. So in this study, we sequenced the sample of *D. Polystachya* based on Jiaozuo and determined its complete chloroplast genome for the first time.

The sample of *D. polystachya* was collected from Jiaozuo, Henan Province (N34°55′25.525″, E113°4′30.385″) on 1 June 2020. The voucher specimen (SY20200603) was deposited in Laboratory of The fourth hospital of Changsha, Changsha (Na Hu, Email: huna202007@163.com). We used the fresh leaves to extract total genomic DNA with the modified CTAB method (Doyle and Doyle 1987) and constructed the libraries with an average length of 350 bp using the NexteraXT DNA Library Preparation Kit (Illumina, San Diego, CA), then the libraries were sequenced on Illumina Novaseq 6000 platform, 2.81 Gb clean data were assembled with de novo assembler SPAdes version 3.11.0 software (St Petersburg, Russa) with default setting (Bankevich et al. 2012) and NOVOPlasty version 2.7.2 (Brussels, Belgium) (Dierckxsens et al. 2016) with kmer (K-mer = 31–33). During the assembling, the complete chloroplast of *Dioscorea nipponica* (MT906794) was used as reference. Finally, the assembled complete cp genome was annotated by PGA software (Qu et al. 2019), and submitted to GenBank under the accession number of MT712160, and SRA submitted to NCBI under the BioProject No. PRJNA694146 and SRA number: SRR13509354,

The total length of complete cp genome of *D. polystachya* is 152,958 bp, with a total GC content of 37.1%. The complete cp genome has a typical quadripartite structure, including a large single-copy (LSC) region of 83,152 bp, a small single-copy (SSC) region of 18,822 bp and two inverted repeat (IRs) regions of 25,492 bp. The complete cp genome contains 131 genes, including 85 protein-coding genes, 38 tRNA, and. 8 rRNA genes. *trn*K-UUU*, rps*16*, trn*G-UCC*, atp*F*, rpo*C1*, trn*L-UAA*, trn*V-UAC*, pet*B*, pet*D*, rpl*16*, rpl*2*, ndh*B*, trn*I-GAU*, trn*A-UGC, and *ndh*A genes contained an intron, and *clp*P and *ycf*3 contained 2 introns.

To determine the phylogenetic position of *D. polystachya*, the complete chloroplast genome of *D. polystachya* was aligned with other 15 species in *Dioscorea* from GenBank using Mafft-7.037 (Katoh and Standley 2013). Subsequently, the phylogenetic tree was constructed by IQTREE version 1.6 (Vienna, Austria) (Nguyen et al. 2015; Hoang et al. 2018) with 1000 bootstrap replicates using Best-fit model. By using *Campynema lineare* as out group, we got the final result. Based on the result (Figure 1), we can find that *D. polystachya* has closely relationship with *Dioscorea alata* (MG267382) and *Dioscorea aspersa* (NC039807). This study was the first time to clearly identify the complete chloroplast of *D. polystachya* base on Jiaozuo and can provide useful information to further study.

**Figure 1.**
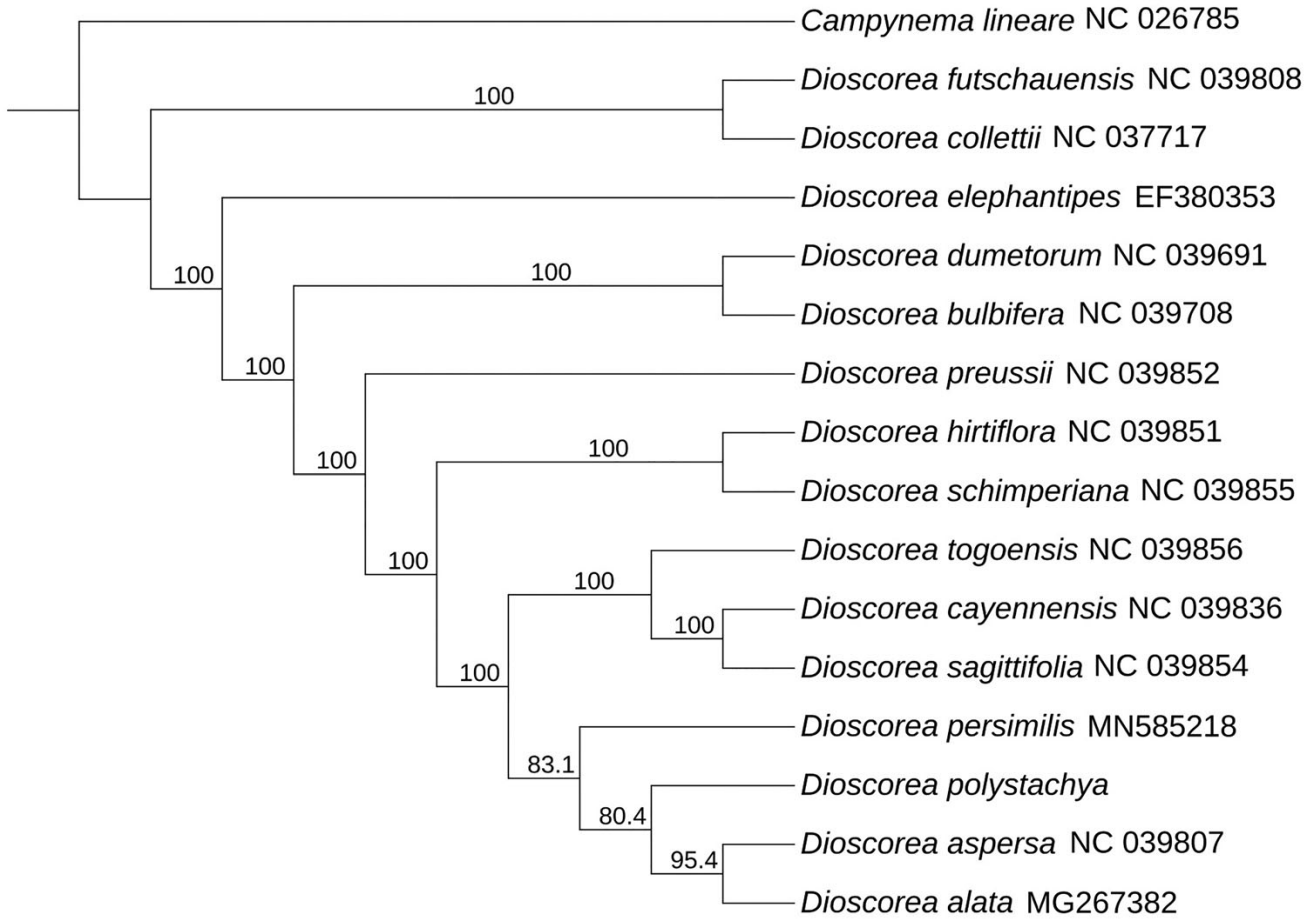
Maximum-likelihood phylogenetic tree for *Dioscorea polystachya* based on 16 complete chloroplast genomes.

## Data Availability

The genome sequence data that support the findings of this study are openly available in GenBank of NCBI at (https://www.ncbi.nlm.nih.gov/) under the accession no. MT712160. The associated BioProject, SRA, and Bio-Sample numbers are PRJNA694146, SRR13509354, and SRS8097807, respectively.
